# Attenuated adenomatous polyposis with *MSH6* variation: Two case reports

**DOI:** 10.1097/MD.0000000000038791

**Published:** 2024-07-05

**Authors:** Gi Won Ha, Min Ro Lee, Ae Ri Ahn, Myoung Ja Chung, Kyoung Min Kim

**Affiliations:** a Department of Surgery, Jeonbuk National University Medical School, Research Institute of Clinical Medicine of Jeonbuk National University, and Biomedical Research Institute of Jeonbuk National University Hospital, Jeonju, Republic of Korea; b Departments of Pathology, Jeonbuk National University Medical School, Research Institute of Clinical Medicine of Jeonbuk National University, Biomedical Research Institute of Jeonbuk National University Hospital, and Research Institute for Endocrine Sciences, Jeonju, Republic of Korea.

**Keywords:** attenuated adenomatous polyposis, *MSH6*, next generation sequencing

## Abstract

**Rationale:**

Adenomatous polyposis (AP) is a genetic disorder characterized by the occurrence of numerous adenomatous polyps in the colon and rectum and can be classified into classical AP and attenuated AP (AAP). AAP is diagnosed when the number of observed adenomas is between 10 and 99. The detection of AAP is significantly increasing mainly due to the improvement of the imaging technique and application of the screening program for colorectal cancer detection. Currently, the germline variations of the *APC* and *MUTYH* genes are reported as the main cause of classical AP. However, the underlying genetic basis of AAP is not well understood. In this study, we report 2 cases of AAP with *MSH6* variations.

**Patient concerns:**

Both patients visited the hospital after multiple polyps were detected during colonoscopies conducted as part of their health checkups.

**Diagnoses:**

The 2 patients were diagnosed with AAP through colonoscopic examination at our hospital.

**Interventions:**

The 2 received genetic consultation; and, for follow-up purposes, both patients agreed to be tested for an underlying genetic condition through next generation sequencing. And germline *MSH6* variations were detected in both AAP patients.

**Outcomes:**

There was no recurrence for both patients for 3 years follow-up.

**Lessons:**

Minor portion of AAP can cause by genetic mutation in *MSH6,* and further research is needed.

## 1. Introduction

Adenomatous polyposis (AP) is a genetic disorder characterized by occurrence of numerous adenomatous polyps in the colon and rectum^.[1]^ AP is classified as classical AP (CAP) and attenuated AP (AAP) according to the number of adenomas.^[1]^ CAP is diagnosed when more than 100 adenomas are present; AAP is diagnosed if the number of adenomas is between 10 and 99.^[2]^ An adenoma is a benign tumor. However, an adenoma is considered to be a precursor of colorectal carcinoma (CRC); these polyps can progress into cancer if untreated.^[3]^ Therefore, the presence of AP is treated as a cancer risk syndrome with the risk of developing CRC ranging from 40% to 80% based on the severity of the polyposis.^[1]^ Currently, a germline heterozygous truncating variation of the tumor suppressor gene *APC* is considered to be the main cause of CAP.^[1]^ In addition, germline biallelic variations of the *MUTYH* gene have been reported to contribute to the development of CAP.^[1]^ However, variations of *APC* and *MUTYH* are the cause of AAP in only 10% to 20% of cases.^[1]^ The *MSH6* gene functions in DNA mismatch repair (MMR).^[4]^ Variation of the *MSH6* gene leads to dysfunction of DNA MMR and is associated with Lynch syndrome.^[5]^ Although *MSH6* variation has been reported in serrated polyposis,^[6]^ this genetic variation has not been reported in AAP. In this study, we report 2 cases of AAP with *MSH6* variation.

## 2. Case presentation

### 2.1. Case 1

**Figure 1.**
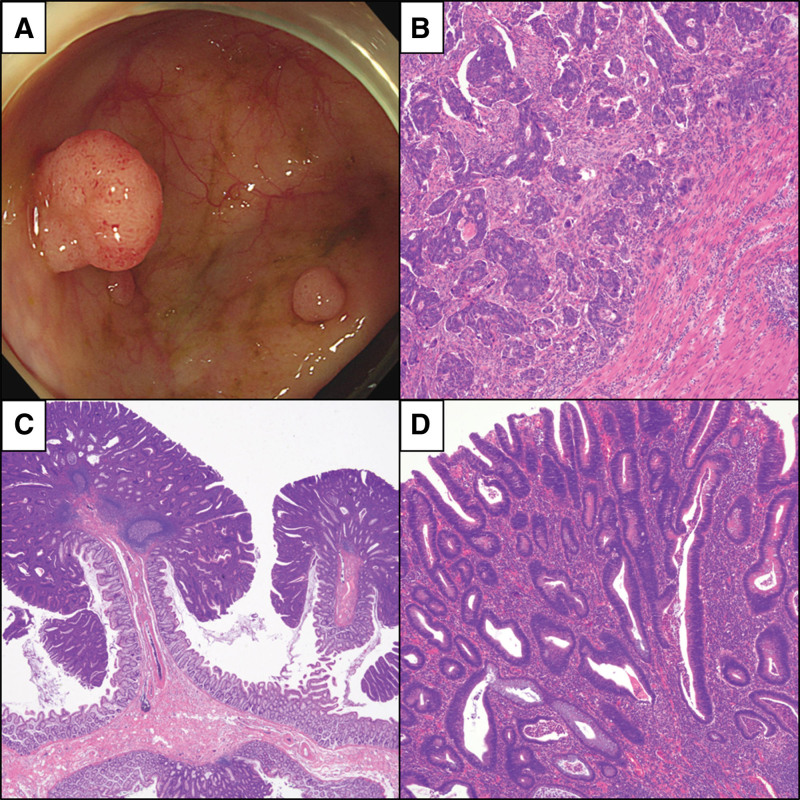
(A) Endoscopy of the colon revealed multiple polyps. (B) Histologic features of the patient’s colon cancer. Tumor cells are infiltrating the smooth muscle layer (H&E stain, magnification: ×100). (C) Low-power view of the colon polyps (H&E stain, magnification: ×20). (D) The polyps show tubular glands with dysplasia (H&E stain, magnification: ×100). H&E = hematoxylin and eosin stain.

A 50-year-old female presented for colonoscopy as part of a medical examination program at a local hospital. More than 20 polyps and a 2 cm mass at the sigmoid colon were identified. The patient was referred to Jeonbuk National University Hospital. After admission, computed tomography was performed, and 2 cm wall thickening of the sigmoid colon was diagnosed. Colonoscopy was also performed, and the sigmoid colon mass with multiple polyps is shown (Fig. 1A). Computed tomography of the other sites showed no metastatic lesions. The sigmoid colon mass was biopsied and diagnosed as adenocarcinoma. The patient had approximately 25 colon polyps, suggesting AAP. Since there was sigmoid colon cancer along with AAP, total colectomy was performed. The sigmoid colon mass was diagnosed as moderately differentiated adenocarcinoma that had metastasized to one regional lymph node (pT2N1a) (Fig. 1B). The colon polyps were diagnosed as tubular adenomas (Fig. 1C, D).

Based on the presence of multiple polyps in the colon, we recommended genetic analysis to the patient. The patient consented, and we performed next-generation sequencing (NGS) with an Oncomine Comprehensive Assay Plus (Thermo Fisher Scientific, Waltham, MA) panel. The result revealed variation in the *MSH6* gene (c.3163G>A, p.Ala1055Thr). No variation was present in the *APC* gene. Since the findings were consistent with AAP, normal tissue was also sequenced through NGS. The identical *MSH6* gene variant (c.3163G>A, p.Ala1055Thr) was detected in the normal tissue. Furthermore, the same *MSH6* variant (c.3163G>A, p.Ala1055Thr) detected in normal and tumor tissues was also found by NGS testing of DNA isolated from patient’s blood sample. Based on these findings, we concluded that the *MSH6* gene (c.3163G>A, p.Ala1055Thr) variation was of germline origin.

### 2.2. Case 2

**Figure 2.**
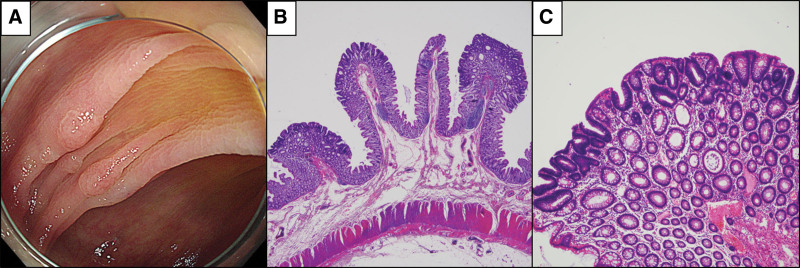
(A) Colonoscopic evaluation showing multiple polyps. (B) Histologic features of the colon polyps. Low-power view of the colon polyps (Hematoxylin and eosin stain, magnification: x20). (C) The polyps show tubular glands with dysplasia (Hematoxylin and eosin stain, magnification: x100). H&E = hematoxylin and eosin stain.

A-53-year-old male had a colonoscopy as part of a medical examination program at a local hospital. Multiple colon polyps were observed on colonoscopic examination, and the patient was referred to Jeonbuk National University Hospital for further evaluation. Abdominal computed tomography and colonoscopic evaluation were performed. Abdominal computed tomography scans showed no abnormalities such as mass. Colonoscopy revealed the presence of approximately 20 polyps (Fig. 2A). Based on the number of colon polyps, the patient was diagnosed with AAP. The size of the largest polyp was 8 mm. Although no cancer was found, the size of this polyp indicated the need for total colectomy. Macroscopically, multiple polyps were identified; and all polyps were diagnosed as tubular adenoma (Fig. 2B, C).

We recommended genetic evaluation to the patient, and the patient consented. We performed NGS panel testing with an Oncomine Comprehensive Assay Plus (Thermo Fisher Scientific, Waltham, MA) panel using normal tissue from the patient. The results showed variation in the *MSH6* gene (c.3163G>A, p.Lys1358AspTer). The *APC* gene was unaltered. Also, the identical *MSH6* variation (c.3163G>A, p.Lys1358AspTer) was detected by sequencing the blood sample of the patient. Since the NGS was performed with normal tissue and blood sample of the patient, the variation in the *MSH6* gene was concluded to be the result of germline variation. The patient had 2 children on whom NGS of was performed using the blood samples and showed the same genetic *MSH6* variant (c.3163G>A, p.Lys1358AspTer), and it was suggested that the variant was inherited from their father.

## 3. Discussion and conclusion

Detection of AAP is significantly increasing.^[1,2]^ The increase of AAP incidence is mostly due to the improvement of imaging and application of screening programs for colorectal cancer detection.^[1]^ AAP is diagnosed when there are more than 10 and less than 100 adenomas in the colon and rectum.^[2]^ AAP patients are a highly heterogenous group regarding severity of disease, family history, and risk for development of CRC. The burden of adenoma can be mild to severe, and risk for development of CRC ranges from 40% to 80% depending on this burden.^[2]^ Age at diagnosis is variable, but AAP patients are generally older than CAP patients.^[2]^ In contrast to CAP, family history of AAP is not a common finding.^[2]^

Variations in *APC* and *MUTYH* genes have been reported to cause CAP.^[1]^ Heterogenous variation of the tumor suppressor gene *APC* resulting in truncation of the protein is the main cause of CAP, and CAP caused by *APC* variation shows a dominant inheritance pattern.^[1]^ In addition, biallelic variations in the *MUTYH* gene, which is involved in DNA repair, causes a minority of CAP cases.^[1]^ However, <20% of AAP cases are caused by variations in the *APC* and *MUTYH* genes; the genetic variants that may be responsible for a significant number of AAP cases are unknown.^[1]^ Studies to elucidate the underlying genetic alterations associated with AAP are ongoing. With the recent development of sequencing technology, new genetic alterations that cause AAP are being identified.^[7–10]^ Other than *APC* and *MUTYH* genetic alterations, variations in *POLE, POLD1, NTHL1, MSH3,* and *MLH3* genes have been reported in AAP cases.^[1]^

In our report, germline variation in the *MSH6* gene was detected in both AAP patients. MSH6 functions in DNA MMR.^[11]^ The protein product hMSH6 combines with hMSH2, the protein product of the *MSH2* gene, and recognizes replication errors in microsatellite sequences.^[11]^ Mutations in MMR genes lead to deficient function in DNA MMR and can cause hereditary non-polyposis colorectal cancer syndrome (Lynch syndrome).^[12]^ Although there is a report of *MSH6* variation in serrated polyposis, to the best of our knowledge, variations of the *MSH6* gene have not been reported in colorectal polyposis syndromes such as CAP and AAP.

*MSH3* encodes hMSH3, an alternative hMSH2 binding partner to hMSH6. hMSH2 has to be combined with hMSH3 or hMSH6 in order to exercise its function. In recent reports, 2 AAP patients were identified to have biallelic truncating variations in the *MSH3* gene.^[9]^ The clinical significance of the *MSH6* mutations detected in our AAP patients is unknown. However, since *MSH3* and *MSH6* share functions, and mutations in *MSH3* were observed in previous AAP patients, deficiencies in MMR may affect the occurrence of AAP as well as the well-known occurrence of hereditary non-polyposis colorectal cancer.

In conclusion, we report 2 cases of AAP with germline *MSH6* variation. Further research is needed to clarify the significance of *MSH6* variation in AAP patients.

## Author contributions

**Conceptualization:** Gi Won Ha, Min Ro Lee.

**Project administration:** Min Ro Lee.

**Writing – original draft:** Min Ro Lee, Kyoung Min Kim.

**Writing – review & editing:** Min Ro Lee, Kyoung Min Kim.

**Data curation:** Ae Ri Ahn, Myoung Ja Chung, Kyoung Min Kim.

**Investigation:** Ae Ri Ahn.

**Methodology:** Myoung Ja Chung, Kyoung Min Kim.
